# The association between autonomy-supportive coaching and athletes’ personal best performance: indirect associations involving basic psychological needs and autonomous motivation

**DOI:** 10.3389/fspor.2026.1803833

**Published:** 2026-06-25

**Authors:** Mingrun Gao, Daliang Zhao

**Affiliations:** 1 Sports Business School, Guangzhou Sport University, Guangzhou, China; 2 School of Sport and Health, Guangzhou Sport University, Guangzhou, China; 3 Guangdong Province Key Laboratory of Human Sports Performance Science, Guangzhou Sport University, Guangzhou, China

**Keywords:** autonomous motivation, autonomy-supportive coaching, basic psychological need satisfaction, personal best performance, self-determination theory

## Abstract

**Introduction:**

Although autonomy-supportive coaching has been widely recognized as beneficial for athletes' motivation and performance, associations between autonomy-supportive coaching and athletes' personal best performance remain insufficiently understood. Grounded in Self-Determination Theory (SDT), this study examined the associations among autonomy-supportive coaching, basic psychological need satisfaction, autonomous motivation, and athletes' personal best performance, within a theory-informed associative framework.

**Methods:**

A total of 411 provincial-level athletes from multiple sports in China completed validated questionnaires assessing perceived coach autonomy support, basic psychological need satisfaction, and autonomous motivation. Athletes' personal best performance was operationalized using official national competitive grading standards and transformed into a continuous indicator. Structural equation modeling with bootstrapping procedures (5,000 resamples) was employed to examine and compare competing models.

**Results:**

The results showed that coach autonomy support, psychological need satisfaction, and autonomous motivation were each positively associated with athletes' personal best performance. Inferential conclusions regarding the two indirect associations were based exclusively on unstandardized indirect effect estimates (B) and their bootstrapped confidence intervals, both of which indicated statistical significance. No statistically significant direct association between coach autonomy support and personal best performance was observed; however, statistically significant indirect associations consistent with the proposed theoretical framework were identified.

**Discussion:**

Although these findings are consistent with the SDT framework, the cross-sectional design precludes causal or temporal inference. All results should therefore be interpreted as reflecting patterns of association. The study offers theoretical and practical implications regarding associations between coaching practices and long-term athletic achievement.

## 1 Introduction

Self-Determination Theory (SDT) posits that social environments are associated with individuals’ motivation in relation to the satisfaction of basic psychological needs: autonomy, competence, and relatedness (1). These needs, when fulfilled, are associated with higher levels of motivation and performance across various achievement contexts (2). In the domain of competitive sports, coaches are considered a central component of autonomy-supportive environments, which is associated with more favorable psychological outcomes and athletic performance (3).

Accumulating evidence indicates that autonomy-supportive coaching is positively associated with the satisfaction of athletes’ basic psychological needs. For example, using adult athlete samples, Adie et al. (4) reported that autonomy-supportive coaching behaviors were positively correlated with athletes’ perceived autonomy, competence, and relatedness. Subsequent research has further identified basic psychological need satisfaction as closely associated with autonomous motivation. In physical education contexts, Haerens et al. (5, 6) examined the associations among perceived autonomy support, need satisfaction, and autonomous motivation, confirming that perceived autonomy-supportive behaviors are positively associated with autonomous motivation and that need satisfaction was positively associated with both autonomy support and autonomous motivation, a pattern statistically consistent with SDT's theoretical framework. While these findings are well-established among physical education students, their direct applicability to competitive athletes warrants caution, given the substantially different motivational demands, performance pressures, and achievement goals inherent in elite sport contexts. Building on this line of inquiry, later studies have highlighted the positive associations between autonomous motivation and personal best performance. Koka et al. (7), for instance, found that coaches’ autonomy-supportive behaviors were associated with athletes’ self-determined motivation, and self-determined motivation was positively associated with objectively assessed performance in competitive events.

Beyond individual studies, meta-analytic and systematic review evidence has reinforced the central role of autonomy-supportive coaching within sport psychology research. Ntoumanis et al. (8) reported robust associations between autonomy-supportive environments and both motivation and performance outcomes, while Nichol et al. (9) identified the theorized associative pattern among autonomy-supportive coaching, psychological need satisfaction, and autonomous motivation as a dominant theoretical framework in contemporary coaching research. Despite this growing consensus, associations among autonomy-supportive coaching, motivation-related variables, and personal best performance remain insufficiently examined. In particular, relatively few studies have examined associations among autonomy-supportive coaching, psychological need satisfaction, autonomous motivation, and personal best performance in competitive athletes from an SDT-informed perspective (10).

Moreover, evidence suggests that direct associations between autonomy-supportive coaching and performance indicators are often weak or inconsistent when motivational variables associated with both autonomy-supportive coaching and performance-related outcomes are not considered [e.g., (11, 12)]. For example, Webster et al. (13) reported minimal differences in autonomy-supportive behaviors between winning and non-winning soccer coaches, indicating that performance outcomes may not be directly associated with coaching style alone. Such findings highlight the importance of examining motivational variables that may be statistically associated with autonomy-supportive coaching and performance-related outcomes.

Another important limitation of existing research concerns the operationalization of personal best performance. Prior studies have predominantly relied on short-term or situational indicators, such as single-competition results or seasonal win–loss records [e.g., (7)]. These measures are susceptible to contextual fluctuations and may not adequately reflect athletes’ stable competitive capacity. In contrast, personal best performance represents a cumulative and developmentally meaningful indicator of athletic achievement, being associated with long-term training engagement, psychological resilience, and sustained competitive competence (14). From an SDT perspective, such a long-term outcome is more theoretically aligned with the motivational framework proposed within SDT than are transient performance indicators.

**Figure 1:**
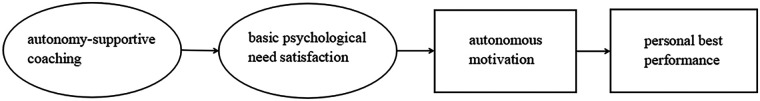
SDT-informed theoretical framework illustrating hypothesized statistical associations among study variables.

Taken together, the key issue concerns associations among autonomy-supportive coaching and competitive performance levels. SDT provides a theoretical framework relevant to examining associations among autonomy-supportive social environments, psychological need satisfaction, autonomous motivation, and performance outcomes (4, 5, 7). Building on this framework, the present study focuses on personal best performance as a long-term performance indicator and examines associations between autonomy-supportive coaching, basic psychological need satisfaction, autonomous motivation, and personal best performance within an SDT-informed framework. This study thereby aims to examine associations between coaching style and long-term competitive achievement within an SDT-informed framework. The findings are expected to contribute associative evidence regarding these variables within an SDT-informed framework and may have implications for evidence-informed coaching practices. The proposed theoretical model is illustrated in Figure 1, and the following hypotheses are advanced:

H1: Autonomy-supportive coaching, basic psychological need satisfaction, and autonomous motivation are positively associated with personal best performance.
H2: Statistically significant associations among autonomy-supportive coaching, psychological need satisfaction, autonomous motivation, and personal best performance are expected, consistent with the SDT-informed theoretical framework.

## 2 Method

### 2.1 Participants

Participants were recruited from a provincial-level sports team in China. A total of 460 questionnaires were initially collected. After data screening, athletes younger than 16 years, those with less than three years of formal training experience, and one Olympic medalist were excluded to reduce potential ceiling effects. The final sample consisted of 411 athletes (48.18% male, 51.82% female), with a mean age of 20.09 years (SD = 3.23) and a mean training experience of 8.90 years (SD = 3.99).

All included participants had competed in at least one sanctioned event that met the official competitive grading standards adopted in the present study. The athletes represented a broad range of sports, including gymnastics, table tennis, fencing, swimming, basketball, trampoline, athletics, water polo, diving, badminton, weightlifting, and tennis. All procedures were approved by the ethics committee of the Guangzhou Sport University, Guangzhou, China.

### 2.2 Measurement

#### 2.2.1 Coach autonomy support

Athletes’ perceptions of coach autonomy support were assessed using the Sport Climate Questionnaire (SCQ), adapted from the Health-Care Climate Questionnaire (HCCQ; (15)). The scale consists of six items (e.g., “I feel that my coach provides me with choices and opportunities”). Participants responded on a 7-point Likert scale, ranging from 1 (strongly disagree) to 7 (strongly agree). The psychometric properties of this instrument have been well-established in previous athletic research [e.g., (16)]. In the present study, the scale demonstrated excellent internal consistency, with a Cronbach's *α* coefficient of 0.926.

#### 2.2.2 Basic psychological needs

Athletes’ basic psychological need satisfaction was measured using the Basic Needs Satisfaction in Sport Scale (BNSSS; (17)). Although the original BNSSS comprises 20 items across five dimensions, the present study employed an adapted version developed through an iterative exploratory factor analysis (EFA) procedure. Prior to EFA, the factorability of the correlation matrix was confirmed: the Kaiser–Meyer–Olkin (KMO) measure of sampling adequacy was 0.923, exceeding the recommended threshold of 0.70 (18), and Bartlett's test of sphericity was statistically significant (*χ*^2^ = 5459.53, df = 136, *p* < 0.001).

EFA was conducted using principal component analysis (PCA) with Varimax (maximum variance method) rotation and Kaiser normalization. Item retention was based on primary factor loadings ≥ 0.40 and the absence of substantial cross-loadings (18). The analysis proceeded through three iterative stages of item removal. First, Item 6 (“I have choices in my training”) was excluded due to cross-loadings on two factors (F1 = 0.523; F4 = 0.561). Second, Item 10 (“I feel I am pursuing my own goals in my training”) was excluded for cross-loadings (F1 = 0.393; F3 = 0.712). Third, Item 14 (reverse-coded: “I do not feel free in my training”) was excluded because its highest loading fell on the Volition factor (0.657) despite reverse-coding, inconsistent with its theoretical assignment. EFA was re-run after each removal until a clean four-factor solution was achieved.

The final EFA solution (17 items) yielded four factors with eigenvalues exceeding 1.0 (8.47, 1.92, 1.63, 1.09; see Supplementary Material 1, Table SM1−2), collectively accounting for 77.14% of the total variance in the rotated solution. All retained items demonstrated clear primary factor loadings ranging from 0.711 to 0.869 with minimal cross-loadings (see Supplementary Material 1, Table SM1−1). The four factors were interpreted as: Competence (Items 1–5; Loadings = 0.719–0.821), Relatedness (Items 16–20; Loadings = 0.711–0.840), Volition (Items 11–13, 15; Loadings = 0.764–0.819), and Choice (Items 7–9; Loadings = 0.858–0.869). The exclusion of the original Novelty subscale (Items 6 and 10) is consistent with prior findings that novelty need satisfaction is less applicable in elite competitive sport contexts (17, 19).

A confirmatory factor analysis (CFA) was subsequently conducted using AMOS 24.0 to evaluate the fit of the final 17-item, four-factor measurement model. The model demonstrated acceptable fit: *χ*^2^(113, *N* = 411) = 311.35, *χ*^2^/df = 2.755, CFI = 0.963, TLI = 0.956, RMSEA = 0.065 [90% CI: 0.057, 0.074]. All standardized factor loadings were statistically significant (*p* < 0.001), ranging from 0.733 to 0.935 (see Material 1, Table SM1−3). Reliability and validity indices are reported in Supplementary Material 1 (Table SM1–4).

Participants responded on a 7-point Likert scale (1 = not at all true; 7 = very true). The four subscales demonstrated good to excellent internal consistency (Cronbach's *α* = 0.87–0.92). As shown in Table SM1–4 (Supplementary Material 1), the adapted BNSSS demonstrated supporting evidence for construct validity. Specifically, all subscales exhibited adequate convergent validity, with Average Variance Extracted (AVE) values ranging from 0.611 to 0.773 (all > 0.50) and Composite Reliability (CR) values ranging from 0.875 to 0.931 (all > 0.70; (18)). Furthermore, discriminant validity was confirmed via the Fornell–Larcker criterion, as the square root of the AVE for each construct (range: 0.782–0.879) was greater than its correlations with all other constructs (range: 0.354–0.746).

#### 2.2.3 Autonomous motivation

Athletes’ motivation was assessed using the Chinese version of the Sport Motivation Scale–II (SMS-II; (20)). Consistent with prior research in Chinese samples, five subscales were retained: intrinsic motivation, identified regulation, introjected regulation, external regulation, and amotivation (21). The structural and convergent validity of this modified instrument have been well-documented within the Chinese athletic context (20).

To quantify the degree of self-determined motivation, the Relative Autonomy Index (RAI) was calculated. Following the established weighting procedure (21, 22), the RAI was computed using the following formula: RAI = (3 × intrinsic) + (2 × identified) - introjected - (2 × external) - (3 × amotivation). Given the high statistical overlap between integrated and identified regulation often observed in sports contexts, integrated regulation was excluded to ensure discriminant validity. A higher RAI score reflects a more autonomous motivational profile. This indexing method is widely recognized as a robust indicator of an athlete's overall self-determined motivation level, particularly within the Chinese athletic population (21, 22).

Although autonomous motivation can be modeled as a latent construct, the present study employed the Relative Autonomy Index (RAI) as an observed composite indicator based on both theoretical and methodological considerations. Within the framework of SDT, the RAI is widely used to index individuals’ overall level of self-determined motivation and provides a parsimonious summary of motivational orientation. Prior research has shown that in Chinese sport samples, the latent structure of autonomous motivation, particularly the distinction between identified and integrated regulation, often exhibits limited discriminant validity, which may relate to unstable factor solutions and inflated correlations when modeled simultaneously (20, 21). In such contexts, composite indices such as the RAI offer a more stable and empirically robust representation of individuals’ overall degree of self-determined motivation. In addition, using the RAI helps reduce model complexity and mitigate multicollinearity among highly correlated regulation types, thereby improving model identification and estimation stability, particularly in structural models including multiple latent constructs and indirect effect analyses. Importantly, the use of the RAI does not replace the theoretical continuum of motivation proposed by SDT; rather, it serves as an empirically grounded summary index of relative autonomy, an approach widely adopted in SDT-based research when examining motivational patterns (42). Accordingly, the use of the RAI in the present study is considered both theoretically justified and methodologically appropriate.

#### 2.2.4 Personal best performance

Personal best performance was defined as the highest competitive achievement attained by an athlete up to the time of data collection. According to official national competitive grading standards, personal best performance was classified into 16 hierarchical levels (23). For analytical purposes, these ordinal categories were transformed into a continuous variable to preserve their hierarchical structure while allowing the estimation of structural equation models.

Specifically, Level 16 (no recorded achievement) was assigned a baseline value of 10.00, with higher levels receiving monotonically increasing scores based on a proportional weighting scheme (see Table 1 for the full scoring scheme). This non-linear scaling reflects the progressively increasing difficulty of attaining higher competitive levels, as the performance gap between adjacent grades grows substantially toward the elite end of the distribution.

**Table 1:** Numerical conversion and scoring of athletes’ personal best (PB) performance.

Rank	Description of competitive achievement	PB score
1	1st place in the Olympic Games	22.82
2	2nd–3rd place in the Olympic Games	18.66
3	1st place in the National Games, World Championships (Olympic events), or World Cup (Olympic events)	16.13
4	4th–8th place in the Olympic Games	14.72
5	2nd–3rd place in the National Games, World Championships (Olympic events), or World Cup (Olympic events)	14.15
6	1st place in the Asian Games (Olympic events)	13.87
7	4th–8th place in the National Games, World Championships (Olympic events), or World Cup (Olympic events)	13.10
8	2nd–3rd place in the Asian Games (Olympic events)	12.89
9	1st place in: Asian Championships/Cup (Olympic events); World Championships/Cup (Non-Olympic events); World Games; Youth Olympic Games; National competitions (National Games events); or Asian Games (Non-Olympic disciplines of Olympic events)	12.32
10	4th–8th place in the Asian Games (Olympic events)	11.62
11	2nd–3rd place in: Asian Championships/Cup (Olympic events); World Championships/Cup (Non-Olympic events); World Games; Youth Olympic Games; National competitions (National Games events); or Asian Games (Non-Olympic disciplines of Olympic events)	11.34
12	1st place in: Asian Championships/Cup (Non-Olympic events); World/Asian Youth Championships (Olympic events); Asian Games (Non-Olympic events); National competitions (Non-National Games events); or National Youth Games	11.06
13	4th–8th place in: Asian Championships/Cup (Olympic events); World Championships/Cup (Non-Olympic events); World Games; Youth Olympic Games; National competitions (National Games events); or Asian Games (Non-Olympic disciplines of Olympic events)	10.85
14	2nd–3rd place in: Asian Championships/Cup (Non-Olympic events); World/Asian Youth Championships (Olympic events); Asian Games (Non-Olympic events); National competitions (Non-National Games events); or National Youth Games	10.56
15	4th–8th place in: Asian Championships/Cup (Non-Olympic events); World/Asian Youth Championships (Olympic events); Asian Games (Non-Olympic events); National competitions (Non-National Games events); or National Youth Games	10.28
16	No recorded achievement	10.00

This transformation was intended to preserve the hierarchical structure of competitive proficiency rather than to impose artificial metric properties. Methodological research suggests that ordinal variables with a large number of categories (typically > 7) can be treated as continuous in SEM with minimal bias in parameter estimates (24); given that the PB measure contains 16 distinct levels, it provides sufficient granularity to approximate such a continuum. Furthermore, to evaluate the robustness of the performance operationalization, the model was re-estimated using the original ordinal grade levels (1–16). The structural coefficients and significance levels remained highly stable across both scaling schemes (see Section 3.5), supporting the invariance of the observed associations. Given the sample size and the complexity of the structural model examining associations among the study variables, this continuous treatment was also deemed more appropriate than ordinal SEM, which typically requires larger samples and stricter distributional assumptions.

### 2.3 Data analysis strategy

Following data screening for validity, preliminary analyses were conducted using SPSS 29.0, including assessments of common method bias (CMB), reliability and validity tests, descriptive statistics, and correlation analyses. Subsequently, structural equation modeling (SEM) was performed using AMOS 24.0 to examine indirect associations between coach autonomy support and personal best performance.

Prior to structural model analyses, the psychometric properties of the adapted BNSSS were evaluated using a two-step approach. First, EFA (PCA with Varimax rotation) was used to empirically identify the factor structure, with item retention based on primary loadings ≥ 0.40 and absence of substantial cross-loadings (18). Second, CFA was conducted using AMOS 24.0 to confirm the measurement structure. Model fit was evaluated against the following criteria: *χ*^2^/df < 5, CFI > 0.90, TLI > 0.90, RMSEA < 0.08 (25). Convergent validity was assessed via average variance extracted (AVE > 0.50) and composite reliability (CR > 0.70; (18)). Discriminant validity was evaluated using the Fornell–Larcker criterion, which requires that the square root of each construct's AVE exceeds its correlations with all other constructs (26).

Internal consistency was confirmed with Cronbach's *α* coefficients for all scales ranging from 0.88 to 0.95. Based on the criteria specified above, all latent constructs demonstrated adequate convergent validity (AVE ranged from 0.56 to 0.72) and discriminant validity (see Fornell–Larcker test results). Detailed psychometric reporting, including the full EFA/CFA procedures, factor loadings, and the discriminant validity matrix, is retained in Supplementary Material 1.

Drawing upon SDT, the study designated coach autonomy support as the independent variable, basic psychological need satisfaction and autonomous motivation as variables included in the theoretically informed association model, and personal best performance as the outcome variable. Ten competing models (Models 1–10) were systematically constructed and compared, including models representing different indirect association specifications among the study variables. This allowed for a rigorous evaluation of the plausibility of different theoretical assumptions.

Model parameters were estimated using the maximum likelihood (ML) method, and the significance of indirect effects was tested using bootstrapping (5,000 resamples). The bootstrapping method was employed because it does not rely on the assumption of normality and provides more robust standard errors and confidence intervals for testing indirect effects (27). The optimal model was selected based on a range of goodness-of-fit indices, with acceptable fit indicated by benchmarks such as *χ*^2^/df < 5, RMSEA < 0.08, and CFI > 0.90, among others (25). The significance of path coefficients (*p* < 0.05) was also considered. The model demonstrating superior fit and consistency with the SDT-informed theoretical framework was retained as the final structural model. Inferential conclusions in the present study are based exclusively on unstandardized indirect effects (B) and their bootstrapped confidence intervals.

## 3 Results

### 3.1 Common method bias assessment

Prior to formal hypothesis testing, confirmatory factor analyses (CFA) and reliability assessments were conducted for each measure. To address potential common method bias (CMB), Harman's single-factor test was performed by subjecting all items of the self-reported scales—including coach autonomy support, basic psychological need satisfaction, and autonomous motivation—to an EFA.

The results revealed five distinct factors with eigenvalues greater than 1. The first unrotated factor accounted for 41.516% of the total variance, which is well below the 50% threshold established in previous literature (28, 29). These findings indicate that common method bias was not a significant concern, and was therefore unlikely to substantially distort the observed associations.

In addition, CFAs indicated that a single-factor model exhibited substantially poorer fit than the hypothesized multi-factor measurement model, providing further evidence that common method variance was unlikely to bias the observed associations.

### 3.2 Descriptive statistics and correlation analysis

Means, standard deviations, and Pearson correlation coefficients for all study variables are presented in Table 2. Descriptive statistics indicated that athletes reported moderate to relatively high levels of perceived coach autonomy support (M = 4.76, SD = 1.17) and basic psychological need satisfaction (M = 4.60, SD = 1.16). The mean levels for autonomous motivation and personal best performance were 2.85 (SD = 8.28) and 12.84 (SD = 2.11), respectively.

**Table 2:** Descriptive statistics and Pearson correlation coefficients among study variables.

Variables	M	SD	1	2	3	4
1 autonomy-supportive coaching	4.76	1.17				
2 basic psychological need satisfaction	4.60	1.16	0.46**			
3 autonomous motivation	2.85	8.28	0.48**	0.64**		
4 personal best performance	12.84	2.11	0.17**	0.17**	0.12*	

M, mean; SD, standard deviation.

* *p* < 0.05.

** *p* < 0.01(two-tailed).

Correlation analyses revealed that coach autonomy support was significantly and positively associated with psychological need satisfaction, autonomous motivation, and personal best performance (*r* = 0.17–0.48, *p* < 0.001). Similarly, psychological need satisfaction exhibited significant positive correlations with autonomous motivation and personal best performance (*r* = 0.17–0.64, *p* < 0.001). Furthermore, autonomous motivation showed a significant positive correlation with personal best performance (*r* = 0.12, *p* < 0.05). These correlation patterns are consistent with theoretical expectations and provide descriptive context for the subsequent indirect association analyses. Additionally, all bivariate correlation coefficients were below the 0.75 threshold, indicating the absence of multicollinearity issues. Overall, these results are consistent with Hypothesis 1 and provide preliminary evidence consistent with Hypothesis 2.

### 3.3 Regression analysis of study variables

Linear regression analyses were performed to evaluate the associations of coach autonomy support, basic psychological need satisfaction, and autonomous motivation with personal best performance (Table 3). As summarized in Table 3, coach autonomy support (*β* = 0.17, *p* < 0.001), psychological need satisfaction (*β* = 0.17, *p* < 0.001), and autonomous motivation (*β* = 0.12, *p* < 0.05) were each significantly associated with personal best performance. These findings correspond to Hypothesis 1.

**Table 3:** Linear regression results for predictors of athletes’ personal best performance.

Predictor variables	*β*	t	R^2^	F	Tolerance	VIF
Coach Autonomy Support	0.17	3.48***	0.03	12.07***	0.73	1.37
Psychological Need Satisfaction	0.17	3.39***	0.03	11.48***	0.56	1.78
Autonomous Motivation	0.12	2.46*	0.02	6.03*	0.55	1.82

β = standardized regression coefficient; *R*^2^ = coefficient of determination; VIF, variance inflation factor.

* *p* < 0.05.

*** *p* < 0.001.

Furthermore, multicollinearity diagnostics revealed that the variance inflation factor (VIF) for all study variables was below 2 (maximum VIF = 1.82). This value is well below the conservative threshold of 5 recommended in methodological literature (18), confirming that multicollinearity was not a concern in the current study and ensuring the stability of the estimated coefficients.

### 3.4 Indirect associations among study variables

Among the ten competing models, selection was based on the joint evaluation of three criteria: statistical fit indices, structural association significance, and consistency with the SDT-informed theoretical framework. Key fit indices for all models are summarized in Table 4; full model descriptions are provided in Supplementary Material 2.

**Table 4:** Fit indices for ten competing structural association patterns.

Model	Associated pathways	*χ* ^2^	df	*χ*^2^/df	RMSEA (90% CI)	CFI	TLI	Selected
1	ASC→NS→AM→PBP	265.043	53	5.001	0.099 [0.087, 0.111]	0.927	0.909	No
2	ASC→NS→PBP	527.991	54	9.778	0.146 [0.135, 0.158]	0.836	0.800	No
3	ASC→AM→PBP	513.368	54	9.507	0.144 [0.133, 0.156]	0.842	0.806	No
**4**	**ASC→NS→AM→PBP; ASC→AM→PBP**	**247**.**887**	**52**	**4**.**767**	**0.096 [0.084, 0.108]**	**0**.**932**	**0**.**914**	**Yes**
5	ASC→NS→AM→PBP; ASC→NS→PBP	262.361	52	5.045	0.099 [0.088, 0.111]	0.927	0.908	No
6	ASC→NS→AM→PBP; ASC→PBP	258.269	52	4.967	0.098 [0.087, 0.110]	0.929	0.910	No
7	ASC→NS→AM→PBP; ASC→NS→PBP; ASC→AM→PBP	245.969	51	4.823	0.097 [0.085, 0.109]	0.933	0.913	No
8	ASC→NS→AM→PBP; ASC→PBP; ASC→NS→PBP	257.563	51	5.050	0.099 [0.088, 0.112]	0.929	0.908	No
9	ASC→NS→AM→PBP; ASC→PBP; ASC→AM→PBP	241.158	51	4.729	0.095 [0.083, 0.108]	0.934	0.915	No
10	ASC→NS→AM→PBP; ASC→NS→PBP; ASC→AM→PBP; ASC→PBP	240.560	50	4.811	0.096 [0.084, 0.109]	0.934	0.913	No

ASC, autonomy-supportive coaching; NS, basic psychological need satisfaction; AM, autonomous motivation; PBP, personal best performance. Acceptable model fit criteria: *χ*^2^/df < 5.0, CFI >0.90, TLI >0.90, RMSEA <0.08 (25). Bold row indicates the selected final model. Model 4 was selected as it was the only model simultaneously meeting the *χ*^2^/df threshold, achieving acceptable CFI and TLI values, and retaining full path significance (*p* < 0.05) across all structural associations. Although Models 9 and 10 showed marginally lower RMSEA values, both contained non-significant structural paths and were therefore excluded.

On statistical grounds, Models 2 and 3 were rejected due to unacceptable fit (*χ*^2^/df = 9.778 and 9.507, respectively), and Models 1, 5, and 8 failed to meet the *χ*^2^/df < 5.0 threshold (*χ*^2^/df = 5.001, 5.045, and 5.050). Among the remaining models with acceptable *χ*^2^/df values (Models 4, 6, 7, 9, and 10), Models 6 and 9 included a non-significant direct association between ASC and PBP, and Models 7 and 10 included a non-significant direct association between NS and PBP; all four were therefore excluded on association significance grounds.

Model 4 was the model that best satisfied the predefined statistical and theoretical criteria: all structural associations were statistically significant (*p* < 0.05), and fit indices (CFI = 0.932, TLI = 0.914, and *χ*^2^/df = 4.767) met predefined thresholds. Although Model 4's RMSEA (0.096 [0.084, 0.108) slightly exceeded the conventional 0.08 threshold, Models 9 and 10—which showed marginally lower RMSEA values (0.095 and 0.096, respectively)—each contained non-significant associations and therefore failed the significance criterion.

On theoretical grounds, Model 4 was also statistically consistent with the SDT-informed theoretical framework, which proposes associations among autonomy-supportive coaching, psychological need satisfaction, autonomous motivation, and performance. In summary, Model 4 was the only model that simultaneously demonstrated (a) acceptable fit across multiple indices (CFI, TLI, *χ*^2^/df), (b) statistical significance of all structural paths, and (c) theoretical coherence with SDT. Accordingly, Model 4 was retained as the final structural model.

To further optimize the model, Model 4 was subsequently refined through re-specification by specifying correlated residuals among specific indicators of coach autonomy support. This modification was guided by modification indices (MI) and was based on the psychometric properties and shared item content of the indicators. Specifically, correlated residuals were specified between items exhibiting substantial content similarity and comparable linguistic phrasing (e.g., items both assessing the provision of choices and the rationale behind training tasks). From a measurement perspective, such items often share common variance—known as local dependency—which is not fully captured by the primary latent construct of autonomy support. By specifying this systematic measurement covariance while preserving the theoretical integrity of the autonomy-supportive coaching construct, the refined model reflected a more accurate representation of the data structure, as recommended by Byrne (30).

**Figure 2:**
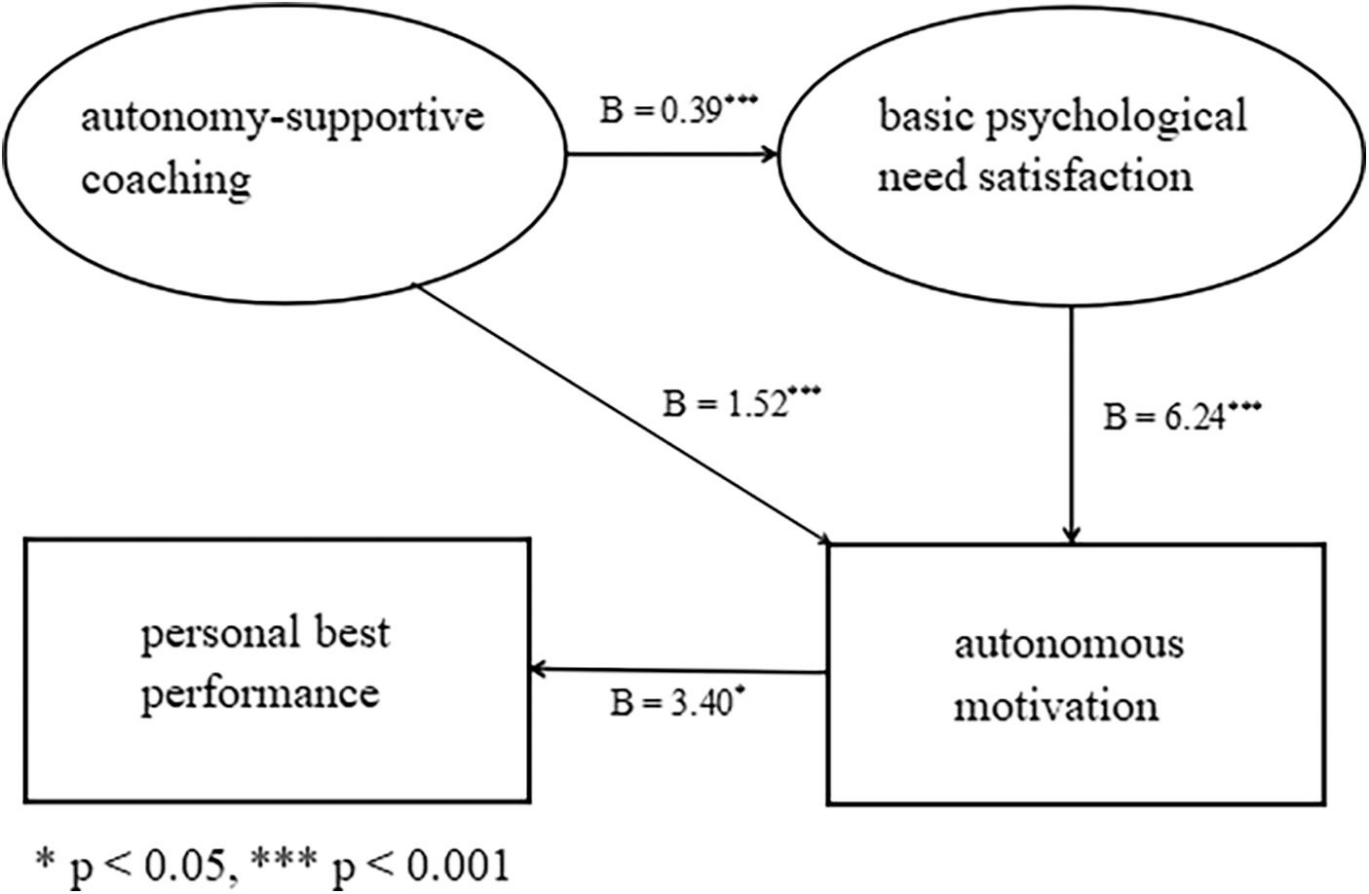
Final SDT-informed associative model (modified model 4).

Following this modification, the model showed substantially improved fit indices compared with those of the original (unmodified) Model 4. A detailed comparison of the fit indices between the initial and modified Model 4 is presented in Table 5. The finalized structural equation model is illustrated in Figure 2.

**Table 5:** Comparison of goodness-of-fit indices between the initial and modified model 4.

Model	*χ* ^2^	df	*χ*^2^/df	RMSEA (90% CI)	CFI	TLI
Initial Model 4	247.887	52	4.767	0.096 [0.084, 0.108]	0.932	0.914
Modified Model 4	151.617	50	3.032	0.070 [0.058, 0.083]	0.965	0.954

Residual correlations were added based on Modification Indices and semantic content overlap.

All structural associations in the modified Model 4 were statistically significant (*p* < 0.05). Coach autonomy support was positively associated with both basic psychological need satisfaction and autonomous motivation. Basic psychological need satisfaction was positively associated with autonomous motivation, and autonomous motivation was positively associated with personal best performance (all *p* < 0.05; see Table 5 for detailed coefficients).

Based on the finalized structural model, two statistically significant indirect associations between coach autonomy support and personal best performance were observed. The first indirect association (M1) was statistically consistent with associations among coach autonomy support, basic psychological need satisfaction, autonomous motivation, and personal best performance. The second indirect association (M2) was statistically consistent with associations among autonomy-supportive coaching, autonomous motivation, and personal best performance. The corresponding indirect effect estimates for these associations are presented in Table 6.

**Table 6:** Indirect effects of pathways in the modified model 4.

Pathway	Unstandardized (B) [Bias-corrected 95% CI]	Standardized (β, descriptive only)
M1: ASC → NS → AM → PBP	8.188 [1.161, 18.639]	0.037
M2: ASC → AM → PBP	5.176 [0.880, 13.321]	0.023

B represents unstandardized indirect effect coefficients with corresponding 95% bias-corrected confidence intervals, which form the basis for statistical inference in this study. Standardized coefficients (β) are reported for descriptive purposes only and are not accompanied by confidence intervals; therefore, they should not be interpreted inferentially or used for comparing effect magnitudes.

As shown in Table 6, the confidence intervals for the unstandardized indirect effects did not include zero, indicating that both indirect association patterns were statistically significant. For the indirect association involving both psychological need satisfaction and autonomous motivation (M1), a statistically significant unstandardized indirect effect was observed (B = 8.188, 95% CI [1.161, 18.639). For the indirect association involving autonomous motivation only (M2), a statistically significant unstandardized indirect effect was also observed (B = 5.176, 95% CI [0.880, 13.321). Both indirect associations were statistically significant based on unstandardized indirect effect estimates (B), with bootstrapped confidence intervals excluding zero. Standardized estimates (*β*) are reported for descriptive reference only and are not used for comparison or inferential interpretation.

### 3.5 Sensitivity analysis

To examine whether the observed associations remained statistically consistent across alternative operationalizations of personal best performance, the primary structural model was re-estimated using the original ordinal grade levels (1–16) as an alternative operationalization of performance. A structured comparison of the model fit indices, structural path coefficients, and indirect effects across both operationalizations is presented in Table 7.

**Table 7:** Sensitivity analysis: comparison across alternative operationalizations of personal best performance.

Fit indices/Path estimates	Proportional scale	Original ordinal scale
Model Fit		
*χ*^2^/df	3.032	3.033
CFI	0.965	0.965
TLI	0.954	0.954
RMSEA [90% CI]	0.070 [0.058, 0.083]	0.070 [0.058, 0.083]
Structural Associations (Standardized β; descriptive only)		
ASC → NS	0.475	0.475
ASC → AM	0.193	0.193
NS → AM	0.643	0.643
AM → PBP	0.121	0.121
Indirect Associations (Unstandardized B)		
M1 (ASC → NS → AM → PBP) [95% CI]	8.188 [1.161, 18.639]	0.058 [0.006, 0.135]
M2 (ASC → AM → PBP) [95% CI]	5.176 [0.880, 13.321]	0.036 [0.005, 0.092]

ASC, Autonomy-supportive coaching; NS, Basic psychological need satisfaction; AM, Autonomous motivation; PBP, Personal Best Performance; CI, confidence interval. Standardized structural coefficients (β) are presented for descriptive purposes only. Inferential conclusions regarding indirect associations are based exclusively on unstandardized indirect effect estimates (B) and their 95% bias-corrected bootstrap confidence intervals. Although unstandardized indirect effect estimates differed numerically because of differences in measurement units, inferential conclusions remained unchanged across operationalizations, with confidence intervals excluding zero for both M1 and M2 under both scaling methods.

As shown in Table 7, the results demonstrate a high degree of consistency across alternative operationalizations of personal best performance. Despite the change in the scaling of the performance variable, all model fit indices remained virtually identical (e.g., CFI = 0.965 and RMSEA = 0.070 for both models). Furthermore, all structural paths retained their direction and statistical significance (*p* < 0.05) across both operationalizations. The association between autonomous motivation and performance remained statistically significant (*p* = 0.014) regardless of the scaling method used.

Regarding indirect associations (see Table 7, Indirect Effect section), both operationalizations yielded statistically significant indirect effect estimates for M1 and M2, with all 95% bias-corrected bootstrap confidence intervals excluding zero. Although unstandardized estimates (B) differed numerically because of differences in scaling units, inferential conclusions remained unchanged across both operationalizations. These results indicate that the observed associations remained statistically consistent across alternative operationalizations of personal best performance, with inferential conclusions remaining unchanged across both scaling approaches.

## 4 Discussion

Grounded in SDT, the present study examined indirect associations between autonomy-supportive coaching and personal best performance. The results indicated that both the indirect association involving psychological need satisfaction and autonomous motivation (M1) and the indirect association involving autonomous motivation only (M2) were statistically significant. Specifically, statistically significant unstandardized indirect effects were observed for both M1 and M2, with 95% bias-corrected confidence intervals excluding zero. Taken together, these findings are statistically consistent with associations among autonomy-supportive coaching, psychological need satisfaction, autonomous motivation, and personal best performance as proposed within an SDT-informed theoretical framework. However, because the present study employed a cross-sectional design, the observed indirect effects should not be interpreted as evidence of causal directionality or temporal ordering.

### 4.1 Correlates of personal best performance

Consistent with SDT, coach autonomy support emerged as positively associated with personal best performance. This finding is consistent with previous studies reporting that autonomy-supportive coaching is positively associated with performance outcomes, a pattern consistent with SDT's theoretical framework (31, 32). Similarly, Gillet et al. (33) reported that autonomy support and autonomous motivation were each positively associated with performance outcomes, a pattern consistent with that observed in the present study.

Basic psychological need satisfaction was also positively associated with personal best performance, a finding statistically consistent with the theoretical framework of SDT (34). Higher levels of autonomy, competence, and relatedness were positively associated with personal best performance, though no causal inference can be drawn from these cross-sectional data. This finding is broadly consistent with prior research reporting positive associations among psychological need satisfaction, motivation-related variables, and performance-related outcomes (8, 35). Autonomous motivation was further positively associated with personal best performance, a finding consistent with extensive evidence that self-determined forms of motivation are associated with higher levels of performance outcomes (36).

### 4.2 Associations between autonomy-supportive coaching and personal best performance

The present study also examined indirect associations between autonomy-supportive coaching and personal best performance. Structural equation modeling results showed that no statistically significant direct association between coach autonomy support and personal best performance was observed; rather, statistically significant indirect associations involving psychological need satisfaction and autonomous motivation were observed, consistent with the proposed theoretical framework. As reported in Section 3.4, statistically significant indirect effects were observed based on unstandardized estimates and bootstrapped confidence intervals.

These findings are statistically consistent with prior SDT-informed research reporting positive associations among psychological need satisfaction, autonomous motivation, and performance outcomes (1). In the present study, both psychological need satisfaction and autonomous motivation showed positive associations with personal best performance within the SDT-informed model. This pattern is consistent with prior research by Gillet et al. (37).

Consistent with SDT's motivational framework, autonomy-supportive coaching was associated with autonomous motivation (3). Greater perceived autonomy-supportive coaching was positively associated with higher levels of psychological need satisfaction in the present sample, a finding consistent with prior SDT-informed research (19, 38, 39). These associations have also been observed across various sport contexts, although their magnitude may vary depending on the specific sport and competitive level (8, 40, 43).

Within the final structural model, autonomous motivation was positively and significantly associated with personal best performance, a finding consistent with prior SDT-based research (2). The association between psychological need satisfaction and personal best performance was not statistically significant in this model. These findings should be interpreted as statistical associations within a cross-sectional model rather than evidence of causal directionality or temporal ordering.

### 4.3 Theoretical and practical implications

The theoretical contributions of the present study are manifold. First, this study provides empirical evidence that autonomy-supportive coaching was statistically associated with personal best performance alongside psychological need satisfaction and autonomous motivation. This finding is consistent with and adds to the empirical literature on SDT in the domain of competitive sports. Second, the theoretical significance of these findings lies in the identification of two distinct indirect associations consistent with an SDT-informed framework: one involving both psychological need satisfaction and autonomous motivation (M1), and another involving autonomous motivation only (M2) (see Table 6 for full results). Statistical inference is based exclusively on unstandardized indirect effects (B) and their bootstrapped confidence intervals; standardized estimates (*β*) are reported descriptively in Table 6. This pattern of findings adds to the theoretical literature on associations among coaching environment, psychological needs, motivation, and performance outcomes as proposed by SDT. Furthermore, the associations observed in the final structural model were statistically consistent with the SDT-informed theoretical framework. Specifically, a non-significant direct association between need satisfaction and performance was observed alongside statistically significant associations among the remaining variables. These findings should not be interpreted as evidence of causal directionality or temporal ordering. Finally, this study adds to the literature on associations between coaching styles and long-term competitive outcomes, offering findings that are inconsistent with the reductionist perspective that elite athletes require only strict control or authoritarian management for competitive achievement.

From a practical perspective, these findings offer associative evidence that may inform athletic coaching and management practices. Perceived autonomy-supportive coaching was positively associated with psychological need satisfaction and autonomous motivation in the present sample, a pattern statistically consistent with the SDT-informed theoretical framework. At the organizational level, these findings are consistent with the potential relevance of autonomy-supportive competencies in coach education programs, given the observed associations between autonomy-supportive coaching and athletes’ psychological need satisfaction and motivation in the present data. Ultimately, these findings are consistent with the value of frameworks that attend to both long-term competitive performance and psychological development as outcomes addressed within the same theoretical framework.

## 5 Limitations and future directions

Despite the contributions of the present study, several limitations should be acknowledged.

First, the sample was drawn exclusively from provincial-level athletes within a single province, which may limit the generalizability of the findings. Athletes from different regions or competitive levels may experience distinct training environments and developmental trajectories, potentially related to differences in the observed associations among the studied variables. Future studies are therefore encouraged to expand the sampling scope across multiple regions and performance levels to improve the generalizability of these findings.

Second, a critical limitation concerns the cross-sectional nature and the inherent temporal mismatch in the study design. While personal best performance was recorded as an achievement prior to data collection, the psychological variables were assessed at the time of the survey. As emphasized by Curran et al. (41), longitudinal designs are required to examine temporal ordering among variables within SDT-based frameworks. Future research should therefore adopt multi-wave longitudinal designs to examine these associations more robustly and to capture longitudinal variation in athlete motivation and performance. Moreover, alternative temporal or directional orderings among the observed variables remain plausible and cannot be ruled out using the present cross-sectional design.

Third, although this study was guided by SDT and incorporated both environmental and motivational factors, the current model remains parsimonious. Additional variables, such as personality traits, psychological resilience, or longitudinal performance trajectories, were not included. Future research is encouraged to integrate a broader range of individual and contextual factors to construct a more comprehensive associative framework for understanding personal best performance.

## 6 Conclusion

Grounded in SDT (1), this study collected questionnaire data from 411 provincial-level athletes and employed structural equation modeling combined with bootstrapping procedures to examine indirect associations between coach autonomy support and personal best performance. The findings showed that coach autonomy support, basic psychological need satisfaction, and autonomous motivation were each positively and significantly associated with personal best performance. In addition, no statistically significant direct association between coach autonomy support and personal best performance was observed; statistically significant associations involving psychological need satisfaction and autonomous motivation were identified, consistent with the SDT-informed framework. Two statistically significant indirect associations were identified: one involving both psychological need satisfaction and autonomous motivation (M1), and another involving autonomous motivation only (M2). Both indirect associations (M1 and M2) were statistically significant based on unstandardized indirect effect estimates (B) with bootstrapped confidence intervals excluding zero; detailed estimates are reported in Table 6. These findings provide associative evidence regarding statistically significant indirect associations among autonomy-supportive coaching, psychological need satisfaction, autonomous motivation, and personal best performance within an SDT-informed framework. However, because the present study employed a cross-sectional design, these findings should be interpreted strictly as statistical patterns of association and not as evidence of causal directionality or temporal ordering.

## Data Availability

The original contributions presented in the study are included in the article/supplementary material, further inquiries can be directed to the corresponding author.

## References

[1] Deci EL Ryan RM. The “what” and “why” of goal pursuits: human needs and the self-determination of behavior. Psychol Inq. (2000) 11(4):227–68. 10.1207/S15327965PLI1104_01

[2] Ryan RM Deci EL. Intrinsic and extrinsic motivation from a self-determination theory perspective: definitions, theory, practices, and future directions. Contemp Educ Psychol. (2020) 61:101860. 10.1016/j.cedpsych.2020.101860

[3] Mageau GA Vallerand RJ. The coach–athlete relationship: a motivational model. J Sports Sci. (2003) 21(11):883–904. 10.1080/0264041031000140374 14626368

[4] Adie JW Duda JL Ntoumanis N. Autonomy support, basic need satisfaction and the optimal functioning of adult male and female sport participants: a test of basic needs theory. Motiv Emot. (2008) 32(3):189–99. 10.1007/s11031-008-9095-z

[5] Haerens L Aelterman N Vansteenkiste M Soenens B Van Petegem S. Do perceived autonomy-supportive and controlling teaching relate to physical education students’ motivational experiences through unique pathways? Distinguishing between the bright and dark side of motivation. Psychol Sport Exerc. (2015) 16:26–36. 10.1016/j.psychsport.2014.08.013

[6] Haerens L Vansteenkiste M De Meester A Delrue J Tallir I Vande Broek G. Different combinations of perceived autonomy support and control: identifying the most optimal motivating style. Phys Educ Sport Pedagogy. (2017) 23(1):16–36. 10.1080/17408989.2017.1346070

[7] Koka A Tilga H Põder T Kalajas-Tilga H Hein V Raudsepp L. The role of perceived coaching behaviours on sport performance among female aesthetic group gymnasts. Acta Kinesiologiae Universitatis Tartuensis. (2020) 26:16–32. 10.12697/akut.2020.26.02

[8] Ntoumanis N Ng JY Prestwich A Quested E Hancox JE Thøgersen-Ntoumani C. A meta-analysis of self-determination theory-informed intervention studies in the health domain: effects on motivation, health behavior, physical activity, and health outcomes. Nat Hum Behav. (2021) 15(2):214–27. 10.1080/17437199.2020.1718529 31983293

[9] Nichol A Hall ET Vickery W Hayes PR. Examining the relationships between coaching practice and athlete outcomes: a systematic review and critical realist critique. Int Sport Coach J. (2019) 6(1):13–29. 10.1123/iscj.2017-0105

[10] Howard JL Gagné M Morin AJS Van den Broeck A. Motivation profiles at work: a self-determination theory approach. J Vocat Behav. (2016) 95:74–89. 10.1016/j.jvb.2016.07.004

[11] Bentley MR Mitchell NG Backhouse SH. Coaching behaviors and athlete motivation: a systematic review of the quantitative literature. Int Sport Coach J. (2019) 6(3):346–58. 10.1123/iscj.2018-0095

[12] Ng JY Ntoumanis N Thøgersen-Ntoumani C Deci EL Ryan RM Duda JL. Self-determination theory applied to health contexts: a meta-analysis. Perspect Psychol Sci. (2012) 7(4):325–40. 10.1177/1745691612447309 26168470

[13] Webster CA Hunt K LaFleche M. Are winning coaches more autonomy-supportive? Examining the context of varsity boys’ soccer. J Sport Behav. (2013) 36(2):209–32.

[14] Rocchi MA Guertin C Pelletier LG Sweet SN. Performance trajectories for competitive swimmers: the role of coach interpersonal behaviors and athlete motivation. Motiv Sci. (2020) 6(3):285–96. 10.1037/mot0000156

[15] Williams GC Deci EL. Internalization of biopsychosocial values by medical students: a test of self-determination theory. J Pers Soc Psychol. (1996) 70(4):767–79. 10.1037/0022-3514.70.4.767 8636897

[16] Cheval B Chalabaev A Quested E Courvoisier DS Sarrazin P. How perceived autonomy support and controlling coach behaviors are related to well- and ill-being in elite soccer players: a within-person changes and between-person differences analysis. Psychol Sport Exerc. (2017) 28:68–77. 10.1016/j.psychsport.2016.10.006

[17] Ng JYY Lonsdale C Hodge K. The basic needs satisfaction in sport scale (BNSSS): instrument development and initial validity evidence. Psychol Sport Exerc. (2011) 12:257–64. 10.1016/j.psychsport.2010.10.006

[18] Hair JF Babin BJ Anderson RE Black WC. Multivariate Data Analysis. 8th ed. Boston: Cengage (2018).

[19] Vlachopoulos SP Karavani E. Psychological needs and subjective vitality in exercise: A cross-gender situational test of the needs universality hypothesis. Hell J Psychol. (2009) 6:207–22.

[20] Li C Kawabata M Zhang L. Validity and reliability of the sport motivation scale-II for Chinese athletes. Int J Sport Exerc Psychol. (2018) 16(1):51–64. 10.1080/1612197X.2016.1153130

[21] Xiang MQ. Paths to promote physical exercise and health well-being in adolescents: based on the self-determination theory model. China Sport Science. (2013) 33(8):21–8. 10.16469/j.css.2013.08.009

[22] Zhu J Yin XC. Relationship between perceived autonomy support from significant others and adolescent exercise behavior: based on self-determination theory. Chin J Sports Med. (2017) 36(1):48–55. 10.16038/j.1000-6710.2017.01.009

[23] State Physical Culture and Sports Commission & Ministry of Personnel. (1996). Implementation measures for rewards for athletes and coaches (Ti Ren Zi No. 314).

[24] Rhemtulla M Brosseau-Liard PÉ Savalei V. When can categorical variables be treated as continuous? A comparison of robust continuous and categorical SEM estimation methods under suboptimal conditions. Psychol Methods. (2012) 17(3):354–73. 10.1037/a0029315 22799625

[25] Hu LT Bentler PM. Cutoff criteria for fit indexes in covariance structure analysis: conventional criteria versus new alternatives. Struct Equ Model Multidiscip J. (1999) 6(1):1–55. 10.1080/10705519909540118

[26] Fornell C Larcker DF. Evaluating structural equation models with unobservable variables and measurement error. J Mark Res. (1981) 18(1):39–50. 10.1177/002224378101800104

[27] Hayes AF. Introduction to Mediation, Moderation, and Conditional Process Analysis: A Regression-based approach. 2nd ed. New York: Guilford Press (2017).

[28] Podsakoff PM Organ DW. Self-reports in organizational research: problems and prospects. J Manage. (1986) 12(4):531–44. 10.1177/014920638601200408

[29] Tang DD Wen ZL. Common method bias tests: problems and suggestions. Psychol Sci. (2020) 43(1):215–23. 10.16719/j.cnki.1671-6981.20200130

[30] Byrne BM. Structural Equation Modeling with AMOS: Basic Concepts, Applications, and Programming. 3rd ed. New York: Routledge (2016). 10.4324/9781315757421

[31] Mallett CJ. Self-determination theory: a case study of evidence-based coaching. Sport Psychol. (2005) 19(4):417–29. 10.1123/tsp.19.4.417

[32] Qureshi AK Butt MZI Jamil M. Influence of coaching styles upon players’ performance. Sky Int J Phys Educ Sports Sci. (2022) 6:72–82. 10.51846/the-sky.v6i0.1688

[33] Gillet N Vallerand RJ Amoura S Baldes B. Influence of coaches’ autonomy support on athletes’ motivation and sport performance: a test of the hierarchical model of intrinsic and extrinsic motivation. Psychol Sport Exerc. (2010) 11(2):155–61. 10.1016/j.psychsport.2009.10.004

[34] Deci EL Ryan RM. “Self-determination research: reflections and future directions”. In: Deci EL Ryan RM, editors. Handbook of Self-determination research. Rochester: University of Rochester Press (2002). p. 431–41.

[35] Reeve J Deci EL. Elements within the competitive situation that affect intrinsic motivation. Pers Soc Psychol Bull. (1996) 22:24–33. 10.1177/0146167296221003

[36] Vallerand RJ. “Intrinsic and extrinsic motivation in sport and physical activity: a review and a look at the future”. In: Tenenbaum G Eklund RC, editors. Handbook of Sport Psychology. 3rd ed. Hoboken: Wiley (2007). p. 59–83. 10.1002/9781118270011.ch3

[37] Gillet N Vallerand RJ Rosnet E. Motivational clusters and performance in a real-life setting. Motiv Emot. (2009) 33:49–62. 10.1007/s11031-008-9115-z

[38] Reinboth M Duda JL. Perceived motivational climate, need satisfaction and indices of well-being in team sports: a longitudinal perspective. Psychol Sport Exerc. (2006) 7(3):269–86. 10.1016/j.psychsport.2005.06.002

[39] Wilson PM Mack DE Blanchard CM Gray CE. The role of perceived psychological need satisfaction in exercise-related affect. Hell J Psychol. (2009) 6:183–206.

[40] Mouratidis A Vansteenkiste M Lens W Sideridis G. The motivating role of positive feedback in sport and physical education: evidence for a motivational model. J Sport Exerc Psychol. (2008) 30(2):240–68. 10.1123/jsep.30.2.240 18490793

[41] Curran T Hill AP Ntoumanis N Hall HK Jowett GE. A three-wave longitudinal test of self-determination theory’s mediation model of engagement and disaffection in youth sport. J Sport Exerc Psychol. (2016) 38(1):15–29. 10.1123/jsep.2015-0016 27018555

[42] Howard JL Gagné M van den Broeck A Guay F Chatzisarantis NLD Ntoumanis N. A review and empirical comparison of motivation scoring methods: an application to self-determination theory. Motiv Emot. (2020) 44(4):534–48. 10.1007/s11031-020-09831-9

[43] Gagné M. Autonomy support and need satisfaction in the motivation and well–being of gymnasts. *J Appl Sport Psychol.* (2003) 15(4):372–90. 10.1080/10413200390238031

