# Supplementary material for: The association between autonomy-supportive coaching and athletes’ personal best performance: indirect associations involving basic psychological needs and autonomous motivation
Source: Front Sports Act Living. 2026 Jun 25;8:1803833. doi: 10.3389/fspor.2026.1803833 (PMC13346087; doi:10.3389/fspor.2026.1803833)
Supplement: Supplementary file 1 [file Supplementaryfile1.doc]

**Supplementary Material 1**

Table SM1-1 Rotated Component Matrix for the Adapted BNSSS (17 Items, 4 Factors; N = 411)

| **Item** | **Item Content (BNSSS)** | **F1 Competence** | **F2Relatedness** | **F3Volition** | **F4Choice** |
| --- | --- | --- | --- | --- | --- |
|  | **Factor 1: Competence (Items 1–5)** |  |  |  |  |
| 1 | I can overcome challenges in my training. | 0.719 | 0.234 | 0.360 | 0.091 |
| 2 | I have the technical skills in my training. | 0.813 | 0.215 | 0.171 | 0.140 |
| 3 | I feel I am good at my sport. | 0.815 | 0.218 | 0.281 | 0.167 |
| 4 | I have the opportunity to feel competent in my sport. | 0.821 | 0.257 | 0.274 | 0.151 |
| 5 | I am capable of performing well in my training. | 0.778 | 0.239 | 0.284 | 0.136 |
|  | **Factor 2: Relatedness (Items 16–20)** |  |  |  |  |
| 16 | I feel close to the people in my training. | 0.224 | 0.712 | 0.314 | 0.129 |
| 17 | I care about the people in my training. | 0.250 | 0.711 | 0.240 | 0.040 |
| 18 | People in my training care about me. | 0.095 | 0.830 | 0.134 | 0.196 |
| 19 | I have people I can trust in my training. | 0.279 | 0.748 | 0.194 | 0.070 |
| 20 | I have close relationships in my training. | 0.221 | 0.840 | 0.125 | 0.053 |
|  | **Factor 3: Volition (Items 11–13, 15)** |  |  |  |  |
| 11 | I truly want to participate in my training. | 0.348 | 0.298 | 0.787 | 0.219 |
| 12 | I am doing what I truly want to do in my training. | 0.422 | 0.226 | 0.764 | 0.177 |
| 13 | I voluntarily participate in my training. | 0.326 | 0.246 | 0.819 | 0.136 |
| 15 | I spontaneously participate in my training. | 0.231 | 0.235 | 0.789 | 0.111 |
|  | **Factor 4: Choice (Items 7–9)** |  |  |  |  |
| 7 | I have a say in my training. | 0.114 | 0.051 | 0.111 | 0.858 |
| 8 | I can participate in decisions in my training. | 0.144 | 0.125 | 0.165 | 0.869 |
| 9 | I have opportunities to make decisions in my training. | 0.166 | 0.155 | 0.123 | 0.867 |
|  | **Removed Items (Final EFA loadings < 0.40)** |  |  |  |  |
| 6* | I have choices in my training. [Cross-loading: F1=0.523, F4=0.561] | 0.523 | — | — | 0.561 |
| 10* | I feel I am pursuing my own goals. [Cross-load: F1=0.393, F2=0.712] | 0.393 | — | 0.712 | — |
| 14R* | I feel pressured in my training. [Reverse-coded; ambiguous loading=0.657] | 0.098 | — | 0.657 | — |

Note. Extraction method: Principal component analysis. Rotation method: Varimax with Kaiser normalization (converged in 6 iterations). KMO = 0.923; Bartlett's χ² = 5459.53, df = 136, p < 0.001. Items marked with * were removed. Loadings ≥ 0.40 are considered acceptable; primary loadings are in bold for clarity. F1 = Competence; F2 = Relatedness; F3 = Volition; F4 = Choice.

Note: SPSS output labels factors 1–4 in order of initial eigenvalue; factor labels above reflect theoretical interpretation after rotation.

Table SM1-2 Eigenvalues and Total Variance Explained for the Final Four-Factor EFA Solution

| **Factor** | **Initial Eigenvalue** | **% of Variance** | **Cumulative %** | **Rotated Sum of Squared Loadings** | **% of Variance(Rotated)** | **Cumulative %(Rotated)** |
| --- | --- | --- | --- | --- | --- | --- |
| **1** | 8.473 | 49.839 | **49.839** | 3.891 | 22.889 | **22.889** |
| **2** | 1.915 | 11.265 | **61.105** | 3.535 | 20.794 | **43.683** |
| **3** | 1.632 | 9.600 | **70.705** | 3.173 | 18.667 | **62.350** |
| **4** | 1.094 | 6.434 | **77.139** | 2.514 | 14.789 | **77.139** |

Note. Extraction method: principal component analysis. Rotation method: Varimax (maximum variance method) with Kaiser normalization. Only components with initial eigenvalues ≥ 1.0 were retained. The four retained components collectively explain 77.14% of total variance (rotated solution).

Table SM1-3 Standardized Factor Loadings from the Confirmatory Factor Analysis of the Adapted BNSSS (N = 411)

| **Factor / Item** | **Item Content** | **Std. Loading** | **p** |
| --- | --- | --- | --- |
| **Competence** | | | |
| 1 | I can overcome challenges in my training. | **0.796** | < 0.001 |
| 2 | I have skilled techniques in my training. | **0.796** | < 0.001 |
| 3 | I feel good at my sport. | **0.896** | < 0.001 |
| 4 | I have the opportunity to feel competent in my sport. | **0.912** | < 0.001 |
| 5 | I am capable of performing well in my training. | **0.830** | < 0.001 |
| **Relatedness** | | | |
| 16 | I feel close to others in my training. | **0.779** | < 0.001 |
| 17 | I care about people in my training. | **0.733** | < 0.001 |
| 18 | People in my training care about me. | **0.782** | < 0.001 |
| 19 | I have people I can trust in my training. | **0.782** | < 0.001 |
| 20 | I have close relationships in my training. | **0.829** | < 0.001 |
| **Volition** | | | |
| 11 | I truly want to participate in my training. | **0.935** | < 0.001 |
| 12 | I am doing what I truly want to do in my training. | **0.918** | < 0.001 |
| 13 | I voluntarily participate in my training. | **0.892** | < 0.001 |
| 15 | I spontaneously participate in my training. | **0.762** | < 0.001 |
| **Choice** | | | |
| 7 | I have a say in my training. | **0.766** | < 0.001 |
| 8 | I can participate in decision-making in my training. | **0.871** | < 0.001 |
| 9 | I have opportunities to make decisions in my training. | **0.871** | < 0.001 |

Note. χ²(113, N = 411) = 311.35, χ²/df = 2.755, CFI = 0.963, TLI = 0.956, RMSEA = 0.065 [90% CI: 0.057, 0.074]. All standardized factor loadings are statistically significant at p < 0.001.

Table SM1-4 Reliability, Convergent Validity, and Discriminant Validity of the Adapted BNSSS Subscales

| **Construct** | **n** | **α** | **CR** | **AVE** | **1. Competence** | **2. Relatedness** | **3.**  **Volition** | **4.**  **Choice** |
| --- | --- | --- | --- | --- | --- | --- | --- | --- |
| 1. Competence | 5 | 0.92 | 0.927 | 0.718 | **[0.847]** |  |  |  |
| 2. Relatedness | 5 | 0.88 | 0.887 | 0.611 | 0.617** | **[0.782]** |  |  |
| 3. Volition | 4 | 0.88 | 0.931 | 0.773 | 0.746** | 0.628** | **[0.879]** |  |
| 4. Choice | 3 | 0.87 | 0.875 | 0.701 | 0.417** | 0.354** | 0.448** | **[0.837]** |

Note. α = Cronbach’s alpha; CR = composite reliability; AVE = average variance extracted. Diagonal values (bold, in brackets) are square roots of AVE. Off-diagonal values are standardized inter-factor correlations derived from the CFA. AVE > 0.50 and CR > 0.70 indicate adequate convergent validity (Hair et al., 2018). Discriminant validity is supported when the square root of AVE exceeds all inter-factor correlations in the same row and column (Fornell & Larcker, 1981). All inter-factor correlations significant at p < 0.001.
