# Supplementary material for: The association between autonomy-supportive coaching and athletes’ personal best performance: indirect associations involving basic psychological needs and autonomous motivation
Source: Front Sports Act Living. 2026 Jun 25;8:1803833. doi: 10.3389/fspor.2026.1803833 (PMC13346087; doi:10.3389/fspor.2026.1803833)
Supplement: Supplementary file 2 [file Supplementaryfile2.doc]

**Supplementary Material 2**

To rigorously evaluate alternative theoretical representations of the data, ten competing structural models were systematically constructed and compared. All models shared the same measurement structure and differed only in the specification of structural paths among autonomy-supportive coaching (ASC), basic psychological need satisfaction (NS), autonomous motivation (AM), and personal best performance (PBP). The models are described below, with structural paths denoted as ASC → NS, ASC → AM, ASC → PBP, NS → AM, NS → PBP, and AM → PBP.

Model 1 specified a single serial indirect association pattern: ASC → NS → AM → PBP. This model assumes that autonomy-supportive coaching is associated with personal best performance exclusively within a sequential indirect framework of need satisfaction and autonomous motivation, with no additional direct or indirect pathways.

Model 2 specified a single simple indirect association pattern including need satisfaction only: ASC → NS → PBP. This model assumes the association between autonomy-supportive coaching and personal best performance is fully accounted for by the indirect pattern including need satisfaction, with autonomous motivation excluded from the pathway.

Model 3 specified a single simple indirect association pathway via autonomous motivation only: ASC → AM → PBP. This model assumes the association between autonomy-supportive coaching and personal best performance is fully accounted for by the indirect pattern including autonomous motivation, with need satisfaction excluded.

Model 4 extended Model 1 by adding one additional pathway, yielding two indirect pathways: (1) ASC → NS → AM → PBP (serial indirect association), and (2) ASC → AM → PBP (simple indirect association via autonomous motivation). This model assumes that autonomy-supportive coaching is associated with personal best performance through both the full serial indirect association pattern and a shorter motivational route.

Model 5 extended Model 1 by adding one alternative pathway, yielding two indirect pathways: (1) ASC → NS → AM → PBP (serial indirect association), and (2) ASC → NS → PBP (simple indirect association via need satisfaction). This model assumes that need satisfaction exerts both a sequential role (through autonomous motivation) and an independent direct association with personal best performance.

Model 6 extended Model 1 by adding a direct path from autonomy-supportive coaching to personal best performance, yielding two pathways: (1) ASC → NS → AM → PBP (serial indirect association), and (2) ASC → PBP (direct effect). This model tests whether autonomy-supportive coaching retains a significant direct association with personal best performance beyond its indirect motivational pathways.

Model 7 extended Model 1 by adding two additional pathways, yielding three pathways in total: (1) ASC → NS → AM → PBP, (2) ASC → NS → PBP, and (3) ASC → AM → PBP. This model assumes that autonomy-supportive coaching is associated with personal best performance through all available indirect routes simultaneously.

Model 8 extended Model 6 by adding one further pathway, yielding three pathways: (1) ASC → NS → AM → PBP, (2) ASC → PBP (direct), and (3) ASC → NS → PBP. This model combines the direct effect of autonomy-supportive coaching with mediation through need satisfaction, both directly and sequentially.

Model 9 extended Model 6 by adding one further pathway, yielding three pathways: (1) ASC → NS → AM → PBP, (2) ASC → PBP (direct), and (3) ASC → AM → PBP. This model combines the serial indirect association pattern with both a direct effect and a simple motivational pathway via autonomous motivation.

Model 10 extended Model 7 by adding a direct path from autonomy-supportive coaching to personal best performance, yielding four pathways: (1) ASC → NS → AM → PBP, (2) ASC → NS → PBP, (3) ASC → AM → PBP, and (4) ASC → PBP (direct). This represents the most saturated model, incorporating all possible indirect and direct pathways simultaneously.

Table SM2-1 Comparison of Fit Indices for the Ten Competing Models

|  | χ² | df | χ²/df | RMSEA（90% CI） | CFI | TLI |
| --- | --- | --- | --- | --- | --- | --- |
| Model 1 | 265.043 | 53 | 5.001 | 0.099 [0.087, 0.111] | 0.927 | 0.909 |
| Model 2 | 527.991 | 54 | 9.778 | 0.146 [0.135, 0.158] | 0.836 | 0.800 |
| Model 3 | 513.368 | 54 | 9.507 | 0.144 [0.133, 0.156] | 0.842 | 0.806 |
| Model 4 | 247.887 | 52 | 4.767 | 0.096 [0.084, 0.108] | 0.932 | 0.914 |
| Model 5 | 262.361 | 52 | 5.045 | 0.099 [0.088, 0.111] | 0.927 | 0.908 |
| Model 6 | 258.269 | 52 | 4.967 | 0.098 [0.087, 0.110] | 0.929 | 0.910 |
| Model 7 | 245.969 | 51 | 4.823 | 0.097 [0.085, 0.109] | 0.933 | 0.913 |
| Model 8 | 257.563 | 51 | 5.050 | 0.099 [0.088, 0.112] | 0.929 | 0.908 |
| Model 9 | 241.158 | 51 | 4.729 | 0.095 [0.083, 0.108] | 0.934 | 0.915 |
| Model 10 | 240.560 | 50 | 4.811 | 0.096 [0.084, 0.109] | 0.934 | 0.913 |

Note. Acceptable model fit was indicated by χ²/df < 5.0, RMSEA < 0.08, CFI > 0.90, and TLI > 0.90 (Hu & Bentler, 1999). SRMR is not directly provided by AMOS and is therefore not reported. Models with any non-significant structural path (p > 0.05) were excluded from further consideration.

Based on the goodness-of-fit indices presented in Table SM2-1, Models 4, 6, 7, 9, and 10 met the acceptable threshold for the χ²/df ratio (< 5.0). However, Models 6, 7, 9, and 10 each contained at least one structural path with a p-value exceeding 0.05, indicating that these path coefficients did not reach statistical significance. From a theoretical standpoint, Model 4 is most consistent with the core SDT proposition that autonomy-supportive coaching, psychological need satisfaction, autonomous motivation, and performance are theoretically associated variables, with the pattern of indirect associations specified in Model 4 being the most coherent with SDT's framework. The more saturated models (e.g., Models 7 and 10) introduce additional association paths that lack strong theoretical grounding within SDT and risk overfitting the data. Although Models 9 and 10 showed marginally lower RMSEA values (0.095 and 0.096, respectively) compared with Model 4 (RMSEA = 0.096), both models contained at least one non-significant structural path (p > 0.05) and were therefore excluded on path significance grounds. Consequently, Model 4 was selected as the final structural model, as it was the only model simultaneously achieving acceptable fit across multiple indices, retaining full path significance across all hypothesized structural associations, and remaining fully consistent with SDT's theoretical framework.
